# Genomic profiling of type-1 adult diabetic and aged normoglycemic mouse liver

**DOI:** 10.1186/1472-6823-14-19

**Published:** 2014-03-03

**Authors:** Flávia G Ghiraldini, André B Silveira, Dirk A Kleinjan, Nick Gilbert, Maria Luiza S Mello

**Affiliations:** 1 Department of Structural and Functional Biology, Institute of Biology, University of Campinas (Unicamp), 13083-862 Campinas, SP, Brazil; 2 Laboratory of Molecular Biology, Centro Infantil Boldrini, Campinas, SP, Brazil; 3 Medical Research Council Human Genetics Unit, Institute of Genetics and Molecular Medicine, University of Edinburgh, Edinburgh, UK

**Keywords:** Type-1 diabetes, Aging, Liver, NOD mouse, Gene expression

## Abstract

**Background:**

Hyperglycemia induces chromatin remodeling with consequences on differential gene expression in mouse hepatocytes, similar to what occurs during aging. The liver is the central organ for the regulation of glucose homeostasis and xenobiotic and lipid metabolism and is affected by insulin signaling. The precise transcriptional profiling of the type-1 diabetic liver and its comparison to aging have not been elucidated yet.

**Methods:**

Here, we studied the differential genomic expression of mouse liver cells under adult hyperglycemic and aged normoglycemic conditions using expression arrays.

**Results:**

Differential gene expression involved in an increase in glucose and impaired lipid metabolism were detected in the type-1 diabetic liver. In this regard, *Ppargc1a* presents an increased expression and is a key gene that might be regulating both processes. The differential gene expression observed may also be associated with hepatic steatosis in diabetic mouse liver, as a secondary disease. Similarly, middle-aged mice presented differential expression of genes involved in glucose, lipid and xenobiotic metabolism. These genes could be associated with an increase in polyploidy, but the consequences of differential expression were not as drastic as those observed in diabetic animals.

**Conclusions:**

Taken together, these findings provide new insights into gene expression profile changes in type-1 diabetic liver. *Ppargc1a* was found to be the key-gene that increases glucose metabolism and impairs lipid metabolism impairment. The novel results reported here open new areas of investigation in diabetic research and facilitate the development of new strategies for gene therapy.

## Background

Type-1 diabetes mellitus (T1DM) is an autoimmune disease caused by lymphocyte infiltration in the endocrine pancreas leading to destruction of β-cells and consequently, to hyperglycemia. Cardiovascular ailments, such as heart attack and atherosclerosis [1], increased prevalence of pancreas, colon and liver cancer [2]; retinopathies, nephropathies and skin conditions are examples of secondary complications caused by hyperglycemia that are accelerated when untreated. Aging shares a number of risk factors with diabetes, such as insulin resistance, higher cholesterol concentration and blood pressure, leading to the theory that diabetes promotes a premature and accelerated aging-like phenotype [3].

Although T1DM starts in the pancreas, the liver is a central organ that regulates glucose homeostasis, xenobiotic metabolism and detoxification, steroid hormone biosynthesis and degradation and lipid metabolism [4].

Hyperglycemia promotes chromatin remodeling and increased polyploidy levels in hepatocytes from non-obese diabetic (NOD) mice; these alterations are similar, but not identical, to the changes observed in hepatocytes from old mice [5]. Recently, it has been observed that the alterations in chromatin organization that occur in hepatocytes from hyperglycemic NOD mice might be orchestrated by the NAD^+^-dependent histone deacetylases Sirt1 and Sirt6 [6]. Although these sirtuins were more abundant in the NOD hyperglycemic mice, their activity was unchanged because of limiting levels of NAD^+^, which could promote differences in gene expression patterns [6].

Genomic profiling studies have been conducted to investigate several organs in diabetic-related experimental conditions, including the type-2 obese diabetic mouse model (fat pads, liver and skeletal muscle) [7], the type-1 diabetic NOD mouse model (pancreatic lymph nodes, spleen and peripheral blood cells) [8] and the streptozotocin-induced type-2 diabetic mouse model (liver) [9,10]. However, there have been no studies investigating the gene expression profile of T1DM liver. In this regard, the NOD mouse model presents an advantage over drug-induced diabetes models because the disease in this mouse strain develops spontaneously; additionally, there is no controversy over whether differences in gene expression in the liver are caused by diabetes-induced agents. Moreover, given the similarities between diabetes and aging in mouse hepatocytes, such as increased polyploidy and chromatin remodeling, we also investigated the effect of hyperglycemia and aging on genomic expression patterns in mouse liver.

## Methods

### Animals

NOD/SHILTJ and Balb/c mice were maintained in an animal care facility on a 12-h light/dark cycle and received food and water *ad libitum*. The protocols involving animal care and use were conducted under the guidance issued by the Medical Research Council in Responsibility in the Use of Animals for Medical Research and Home Office Project License PPL 60/3785 (Edinburgh, UK) and by the Committee for Ethics in Animal Use of the University of Campinas, Brazil (registration no. 1608–1).

The glycemia levels of the animals were checked once a week up to 24 h before they were euthanized. Blood samples were obtained by caudal puncture and analyzed using the automatic Accu-Check Performa glucose meter (Roche Diagnostica do Brasil, Jaguare, Brazil). Glycemia levels within the 90–100 mg/dL (5.00-5.55 mmol/L) range were considered normal, and glycemia levels over 500 mg/dL (27.5 mmol/L) for three weeks were considered indicative of severe hyperglycemia. Four mouse groups were used in this study: 1) three normoglycemic Balb/c young-adults (8-weeks-old); 2) three normoglycemic Balb/c mice (47-weeks-old) 3) two severe hyperglycemic NOD adults and a technical replicate, and 4) normoglycemic NOD mice matched for age to the diabetic groups. The technical replicate was obtained mincing fragments of livers from two hyperglycemic NOD mice and processing them as a completely independent sample. For the microarray validation by qPCR five different animals of each group were used.

The animals were euthanized by cervical dislocation, and their livers were removed and frozen in liquid nitrogen for molecular assays.

### RNA preparation

Total RNA was extracted from livers with Tri-Reagent (Sigma®, St. Louis, EUA). Crude RNA samples were On-Column purified with the RNeasy mini kit (Qiagen®, Hilden, Germany) and analyzed with an Agilent 2100 BioAnalyzer (Agilent®, Santa Clara, USA) before being subjected to microarray studies. Only samples with an RNA Integrity Number (RIN) above 7 were further processed.

### cRNA synthesis, labeling and hybridization

Total RNA (500 ng) was used to synthesize cRNA using the Illumina TotalPrep RNA Amplification kit (Illumina®, San Diego, USA) according to the manufacturer’s protocol. Hybridization, washing and scanning of Illumina Whole Genome Mouse WG-6v2 Gene Expression BeadChips were performed according to a standard protocol.

### Microarray analysis

Raw signal intensity measurements, background correction and normalization of samples were processed with the Illumina GenomeStudio software. Unpaired Limma analyzes using the Limma package for the R-based Bioconductor software [11] were used to obtain differentially expressed probesets with p < 0.05 and fold-change >2.0. We obtained two separate differentially expressed gene lists corresponding to the comparison of Balb/c young-adults vs. aged Balb/c mice and the comparison of normoglycemic NOD mice vs. severe hyperglycemic NOD mice. The IPA software (Ingenuity Systems) was used to functionally interpret the lists of differentially expressed genes, and to analyze enrichment of gene ontology groups and regulation of molecular networks. The raw array data are available at the NCBI GEO database with the accession number GSE50613.

### Validation of microarray results by quantitative RT-PCR

Validation of differential gene expression was performed for the *Pck1, Igfbp1, Sirt1, Srebp1, Ppargc1a, Apoe* and *Foxo1* genes by quantitative RT-PCR using the protocol described above. Five animals were used for each experimental condition (Additional file 1). Relative expression was calculated using β-actin as the endogenous control.

## Results

**Figure 1:**
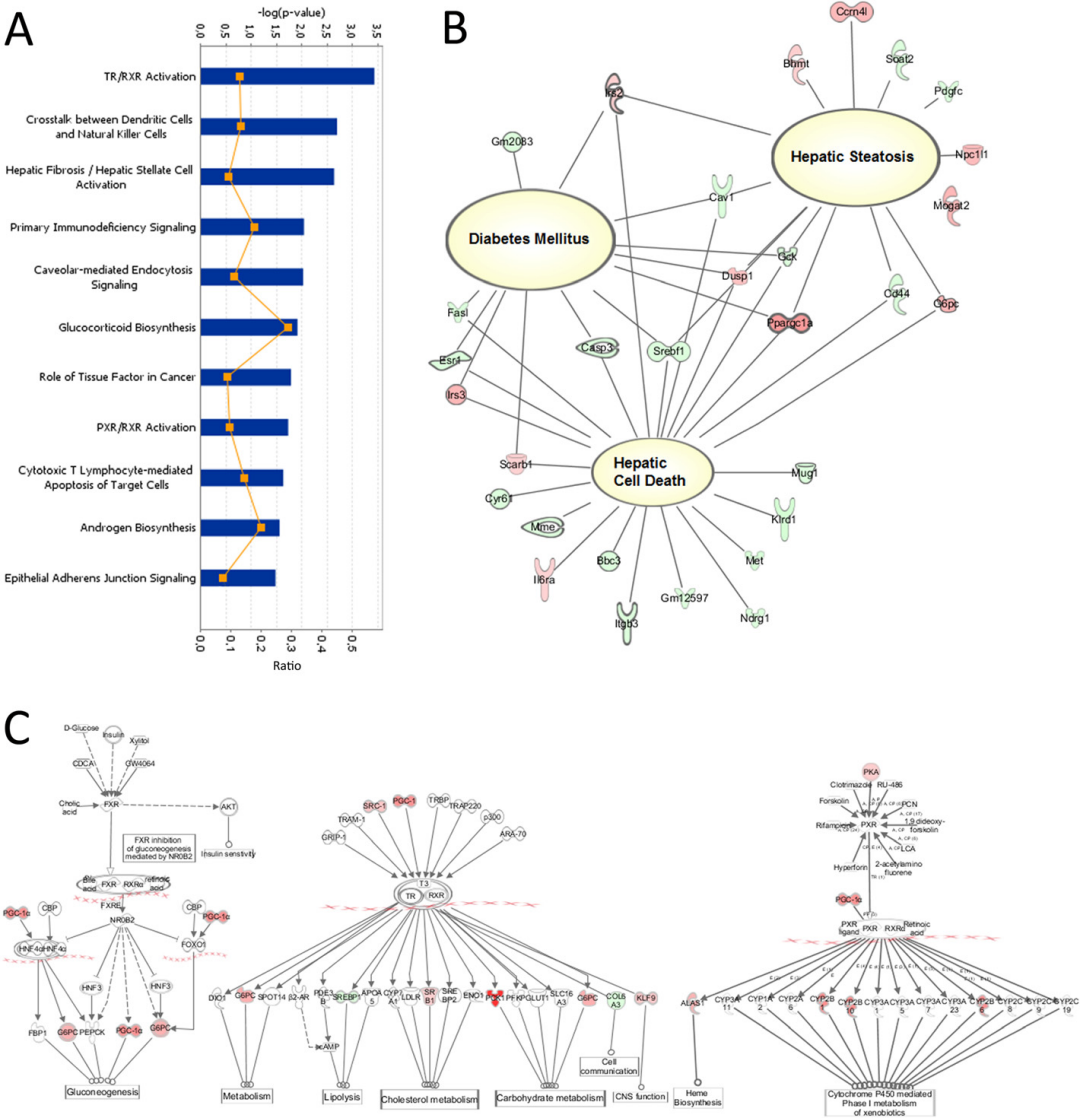
**Diabetic NOD mouse panel. (A)** The most activated canonical pathways in hyperglycemic NOD mice. –log(p-value), the probability that the association between the genes in the dataset and the canonical pathway is due to chance alone; ratio, the number of genes from the dataset that map to the pathway divided by the total number of genes that map to the canonical pathway. **(B)** Differentially expressed genes related to diseases found in the dataset. **(C)** Pregnane Receptor X and Thyroid Receptor canonical pathways. Genes in various shades of red indicate overexpression; genes in various shades of green indicate repression.

Of the 26,766 genes with established sequences available on the Illumina® microarray chip, 219 were found to be differentially expressed (FC > 2.0; p < 0.05) under hyperglycemic conditions in comparison to normoglycemic conditions. Of these differentially expressed genes, 86 were up-regulated, and 133 genes were down-regulated. The full list of differentially expressed genes is presented in Additional file 2. The quantitative PCR using five biological replicates demonstrated the consistency of our global expression profiling results (Additional file 1). We observed that the canonical pathways enriched by hyperglycemia were mainly involved in carbohydrate and lipid metabolism (TR/RXR activation and PXR/RXR activation pathways), as well as in inflammatory signaling (crosstalk between dendritic cells and natural killers cells; primary immunodeficiency signaling; caveolar-mediated endocytosis signaling; cytotoxic T-lymphocyte-mediated apoptosis of target cells) (Figure 1-A). Based on which genes were differentially expressed and their functions, a trend towards metabolic diseases such as hyperglycemia (diabetes mellitus), hepatic steatosis and hepatic cell death (Figure 1-B) could be identified.

**Figure 2:**
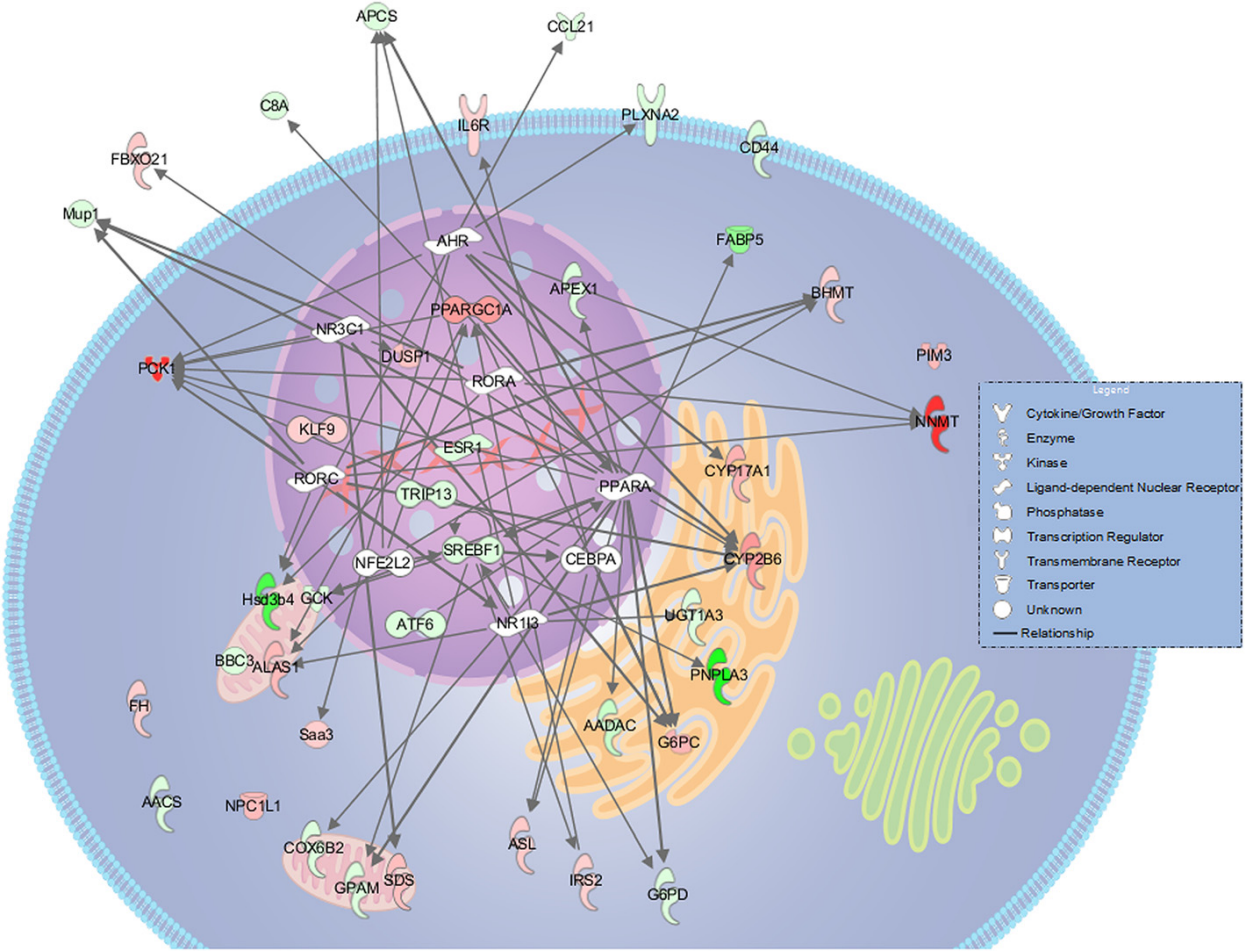
**Metabolic network of diabetic NOD hepatocytes.** Genes in shades of red indicate overexpression; genes in shades of green indicate repression; genes in white were not found to be differentially expressed in our dataset. Arrow, direct relationship.

From these data, *Ppargc1a* (peroxisome proliferator-activated receptor gamma coactivator 1 alpha) was found to be a key regulatory gene playing roles in two canonical pathways that were differentially expressed under hyperglycemic conditions (high p-values): the TR/RXR activation (thyroid hormone receptor) and PXR/RXR activation (Pregnane X receptor) pathways (Figure 1-A). *Ppargc1a* transcribes the transcription factor PGC-1α and is also involved in other pathways with lower p-values. In these pathways, *Ppargc1a* increased gluconeogenesis, carbohydrate and xenobiotic metabolism and repressed lipolysis (Figure 1-C). When the genes were analyzed from a functional perspective, a general decrease in the expression of genes related to lipid metabolism and molecular transport and an increase in the expression of genes involved with carbohydrate metabolism and detoxification were detected; these pathways form a “metabolic network” (Figure 2).

**Figure 3:**
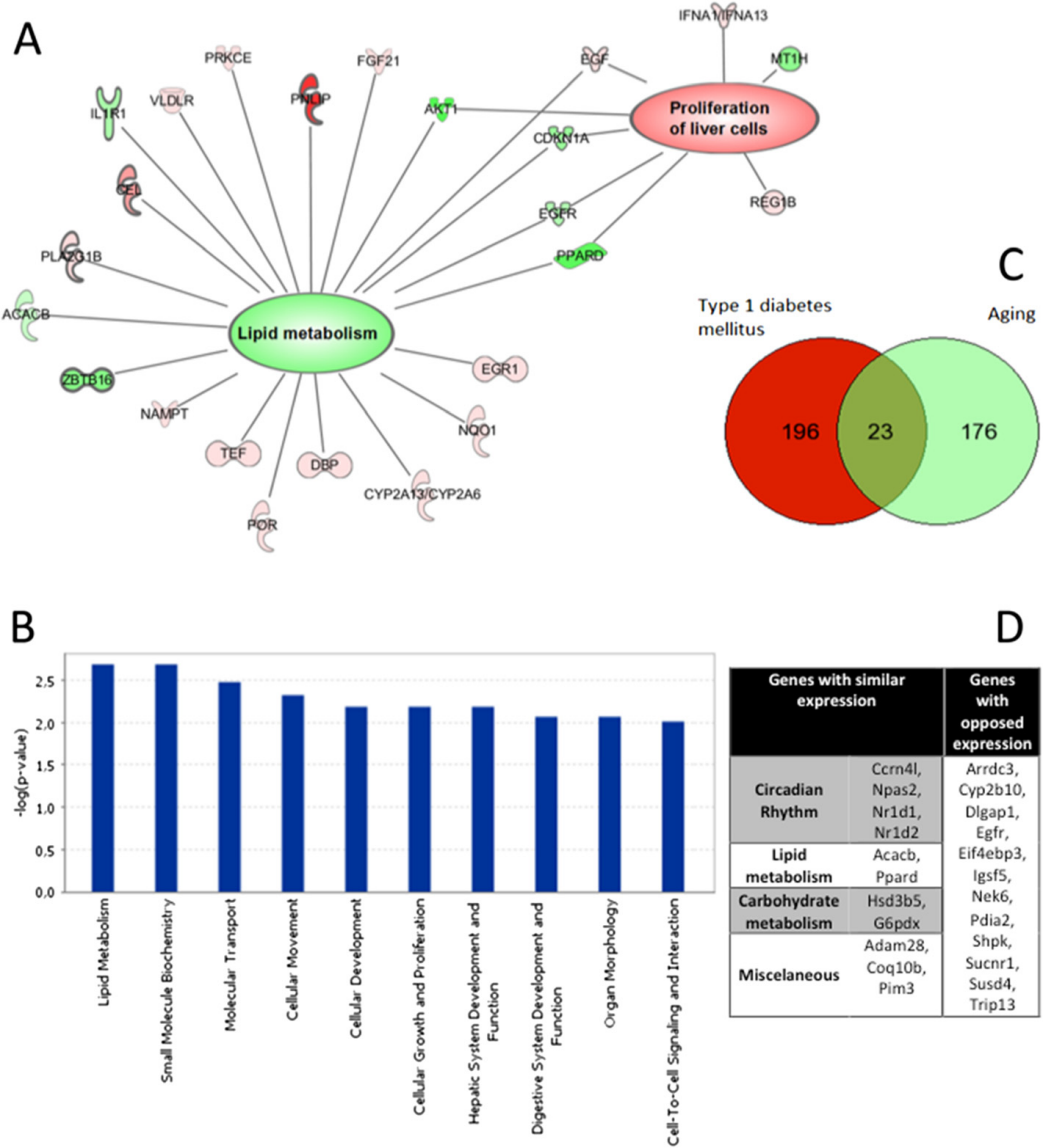
**Middle-aged mice panel. (A)** Differentially expressed genes related to altered biological functions in aging. Genes in shades of red indicate overexpression; genes in shades of green indicate repression. **(B)** The most frequently altered biological function in middle-aged mice hepatocytes. –log(p-value), the probability that the association between the genes in the dataset and the biological function is due to chance alone. **(C)** Venn diagram of genes differentially regulated in both diabetic and middle-aged mouse hepatocytes. **(D)** List of genes with inverted and similar expression between diabetic and middle-aged mice.

The middle-aged mice had 199 differentially expressed genes compared to the young-adult mice; of these, 122 were up-regulated, and 77 were down-regulated. The list of differentially expressed genes is presented in Additional file 3. The global expression profiling and qPCR results yielded similar trends, which can be observed in Additional file 1. Decreased lipid metabolism and increased hepatocyte proliferation were hallmarks of middle-aged mice (Figure 3-A). Among the most significant canonical pathways found differentially regulated in this experimental group, seven pathways indicated up-regulation of genes related to cytochrome P450 (Figure 3-B).

Considering all the genes that were differentially expressed in both experimental groups, a total of 23 genes were shared between adult diabetic and middle-aged normoglycemic mice (Figure 3-C). Twelve of the shared genes were oppositely expressed in the two experimental groups, 11 were similarly expressed and were involved in circadian rhythm, lipid and carbohydrate metabolism (Figure 3-D). The 12 genes with inverted expression levels were related to several functions and could not be grouped into functional categories (Figure 3-D).

## Discussion

Type-1 diabetes mellitus is an autoimmune disease that fundamentally produces an imbalance in carbohydrate and lipid metabolism. The genes that were differentially expressed in hyperglycemic animals played roles in the regulation of both metabolic processes and inflammatory signaling. Additionally, the transcription factor *Ppargc1a* (PGC-1α) was a key upstream regulator. PGC-1α acts with the histone deacetylase Sirt1 as a metabolic sensor in hepatocytes and increases the expression of genes involved in the gluconeogenesis pathway [12]. This study confirmed that PGC-1α was up-regulated in hyperglycemic mice, a finding that has been demonstrated previously at the protein level [6], and highlighted *Pck1* (phosphoenol pyruvate carboxykinase 1) and *G6pc* (glucose −6-phosphatase) as targets of PGC-1α. *Pck1* and *G6pc* are glycolytic genes active in the thyroid receptor pathway and induce gluconeogenesis [13].

PGC-1α also plays a role in lipid metabolism. In the thyroid receptor canonical pathway, this protein induces the expression of *Srb1* (Scavenger receptor B member 1), which increases the uptake of cholesterol esters from high-density lipoproteins (HDL) in the liver [14]. Furthermore, PGC-1α decreases the expression of *Srebp1* (Sterol regulatory element-binding transcription factor 1), inhibiting fatty acid synthesis; this phenomenon has also been observed previously in Zucker diabetic fatty rats [15].

In untreated cases of T1DM and T2DM, fatty liver disease is a common secondary complication [16]. This is caused by an increased internalization of triglycerides in the liver, enhanced hepatic fat synthesis and decreased oxidation [17]. Indeed, we observed that the majority of the differentially expressed genes related to lipid metabolism in the diabetic mouse liver transcribe molecules that facilitate lipid uptake or regulate this process, such as *Npc1l1* (Niemann-Pick C1-like 1), *Srebp1*, *Bhmt* (betaine-homocysteine S-methyltransferase) and *Cav1* (Caveolin 1) [18-20]. The improper balance of genes related to lipid metabolism in diabetic NOD hepatocytes may promote the development of hepatic steatosis as a secondary complication, contributing to the aggravation of T1DM [21]. However, other genes, such as *Srebp1* and *Fabp5* (fatty acid binding protein 5) involved in the cholesterol biosynthesis and uptake, were down-regulated. Therefore, although hepatic steatosis is suggested, no morphological or anatomic alterations could be identified in the samples analyzed. This might have occurred because alterations in the mRNA level could be still preliminary and might need longer period to fully develop into different phenotypes.

Similar to diabetes, middle-aged mice presented differentially expressed genes related to lipid metabolism. When the biological functions of these genes were analyzed, an overall decrease in genes involved in lipid metabolism was identified. The impaired lipid metabolism could be linked to the increased polyploidy previously observed in diabetic and aged mice [5], where a switch from oxidation of fatty acids to oxidation of glucose occurs [17]. This confirm the hypothesis that polyploidization is a response to high metabolic load and stress injury [22]. Oxidative DNA injury, similarly to that observed in T1DM [6] could impair proliferation, although promoting mitogenic stimulation following polyploidy [23]. The polyploidization process could enhance cell survival pathways and differential energy consumption in the liver preferring carbohydrates rather than fatty acids for ATP production [24].

Indeed, the misbalance on lipid and carbohydrate metabolism can be linked to increased production of reactive oxygen species (ROS), highly abundant in untreated cases of T1DM [25]. The ROS generated by excess glucose has been shown to increase DNA fragmentation and cell damage [6], which might contribute to differentially regulate genes involved in stress responses and growth arrest that leads to hepatic cell death. In fact, cytochrome P450 is involved in ROS formation [26]. Increased ROS has been considered as one of the hallmarks of aging and recently regarded as a stress-triggered survival signal [27,28]. As the organism gets older, ROS levels surpass the threshold that the cells could attempt to maintain for normal function [27]. In this study, two subunits of cytochrome P450 complex were found overexpressed in T1DM mice, which might contribute for the increase in DNA damage and be the effector of possible phenotypical changes [6]. The key-genes found here are responsible for the phase 1 of xenobiotic metabolism, when an initial modification and activation of typically lipophylic xenobiotics occur [29]. A counterbalance of four overexpressed and four down-regulated subunits of the cytochrome P450 was observed in aged mouse liver; however there were also 16 overexpressed genes related to stress response. This response might also be a consequence of polyploidy because increased expression of genes involved in stress responses and chaperones occur as a physiological adaptation to aging [17]. Several differentially expressed genes in middle aged-mice were related to hepatocyte proliferation. *Cdkn1a* (p21), an inhibitor of cyclin-dependent kinases [30], *Egf* (Epidermal growth factor) [31] and *Mt1h* (metalloproteinase 1), which is a negative growth regulator [32], are genes involved in polyploidization and cell proliferation and were differentially expressed. During the polyploidization process the mouse hepatocytes may enter in mitosis with incomplete cytokinesis and present not only upregulated genes involved in cell proliferation but also with stress response [24,33] similar to what was observed in this study. This observation indicates a possible trend toward polyploidization during the aging process, which is a well known phenomenon in mouse liver [5,34-36].

In diabetic NOD mice, livers also presented differentially expressed genes involved in immune signaling. The canonical pathways related to inflammation and immune response indicated a role for these genes in innate immunity, antigen presentation and immunodeficiency. The crosstalk between the dendritic cells and natural killer cells pathway has been related in other studies to be one of the responsible alterations found in NOD mice that could elicit T1DM [37,38]. In the liver of Type-1 diabetic NOD mouse, a small population of B220^+^-precursors dendritic cells with strong immune regulatory properties is deleted, confirming that the liver may affect the development of the autoimmunity of diabetes in NOD mice [37,38]. In general, most of the differentially expressed genes in these mice were down-regulated, including the bone marrow stromal cell antigen 2 (*Bst2*), Fas ligand (*Faslg*) and interferon regulatory factor 7 (*Irf7*). A differential expression of these genes has also been observed in Type-2 diabetic mouse hepatocytes [7]. It is known that with aging there is an accumulation of pro-inflammatory tissue damage and that senescent cells secrete pro-inflammatory cytokines, enhancing NF-kB signaling [28]. In this study, middle-aged mice presented 12 genes differentially expressed that were involved with inflammation, the most predominant class of which was represented by chemokines. This indicates that both type-1 diabetic and middle-aged mice presented a misbalance in inflammatory response, although during diabetes it was due to the auto-immune characteristic of the disease.

## Conclusions

Taken together, the present findings provide new insights into gene expression profile changes in type-1 diabetic liver. These alterations have consequences for several biological processes and key genes, such as *Ppargc1a.* This particular gene increases glucose metabolism and impairs lipid metabolism, possibly leading to hepatic steatosis. Moreover, the differential gene expression pattern observed in the middle-aged mouse liver could be associated with polyploidization processes and other physiological adaptations. The novel results reported here highlighted PGC-1α as a key regulator in diabetes and should open new areas of investigation in diabetes research and even promote the development of strategies for gene therapy.

## Abbreviations

Apoe: Apoliprotein E; Bhmt: Betaine-homocysteine S-methyltransferase; Cav1: Caveolin 1; Fabp5: Fatty acid binding protein 5; Foxo1: Forkhead box protein 1; G6PC: Glucose – 6 - phosphatase; HDL: High density lipoprotein; Igfbp1: Insulin growth factor-biding protein 1; NOD mouse: Non-obese diabetic mouse; Pck1: Phosphoenol pyruvate carboxykinase 1; PGC-1α/ Ppargc1a: Peroxisome proliferator-activated receptor gamma coactivator 1-alpha; PXR: Pregnane X Receptor; ROS: Reactive oxygen species; Sirt1: Sirtuin 1; Sirt6: Sirtuin 6; Srb1: Scavenger receptor B member 1; Srebp1: Sterol regulatory element-binding transcriptor factor 1; T1DM: Type-1 diabetes mellitus; T2DM: Type-2 diabetes mellitus; TR: Thyroid hormone receptor.

## Competing interests

The authors declare that they have no competing interests.

## Authors’ contributions

FGG researched data and wrote the manuscript, ABS contributed to the statistical analysis and revised the manuscript, DAK provided the animals in Edinburgh and revised the manuscript, NG contributed to experiment design and revised the manuscript, MLSM contributed to discussion and revised the manuscript. All authors read and approved the final manuscript.

## Pre-publication history

The pre-publication history for this paper can be accessed here:

http://www.biomedcentral.com/1472-6823/14/19/prepub

## Supplementary Material

Additional file 1 **(A) List of the primers used for quantitative gene expression validation. (B)** Gene expression validation. Click here for file

Additional file 2 List of differentially expressed genes in diabetic NOD mouse hepatocytes. Click here for file

Additional file 3 List of differentially expressed genes in middle-aged Balb/c mouse hepatocytes. Click here for file
