# Supplementary material for: Genomic profiling of type-1 adult diabetic and aged normoglycemic mouse liver
Source: BMC Endocr Disord. 2014 Mar 3;14:19. doi: 10.1186/1472-6823-14-19 (PMC4016577; doi:10.1186/1472-6823-14-19)
Supplement: Additional file 1 — (A) List of the primers used for quantitative gene expression validation. (B) Gene expression validation. [file 1472-6823-14-19-S1.docx]

| **Gene** | **Primer *Forward*** | **Primer *Reverse*** |
| --- | --- | --- |
| *ApoE* | AACCGCTTCTGGGATTACCT’ | TTCCGTCATAGTGTCCTCCA |
| *Igfbp1* | AGCCCAGAGATGACAGAGGA | GTTGGGCTGCAGCTAATCTC |
| *B-Actin* | CTAAGGCCAACCGTGAAAAG | CCATCACAATGCCTGTGGTA |
| *Sirt1* | CTCCTGTTGACCGATGGACT | ATCGGTGCAATCATGAGAT |
| *Sirt6* | CCTGTAGAGGGGAGCTGAGA | GAGGTACCCAGGGTGACAGA |
| *Sirt7* | GAGAGCGAGGATCTGGTGAC | GCCCGTGTAGACAACCAAGT |
| *Foxo1* | GCTGGGTGTCAGGCTAAGAG | TTGCCAAGTCTGAGGAAAGG |
| *Pck1* | CCTGGAAGAACAAGGAGTGG | CTACGGCCACCAAAGATGAT |
| *Srebp1* | CCACACTTCATCAAGGCAGA | AGGTACTGTGGCCAAGATGG |
| *Ppargc1a* | AATGCAGCGGTCTTAGCACT | GTGTGAGGAGGGTCATCGTT |

**
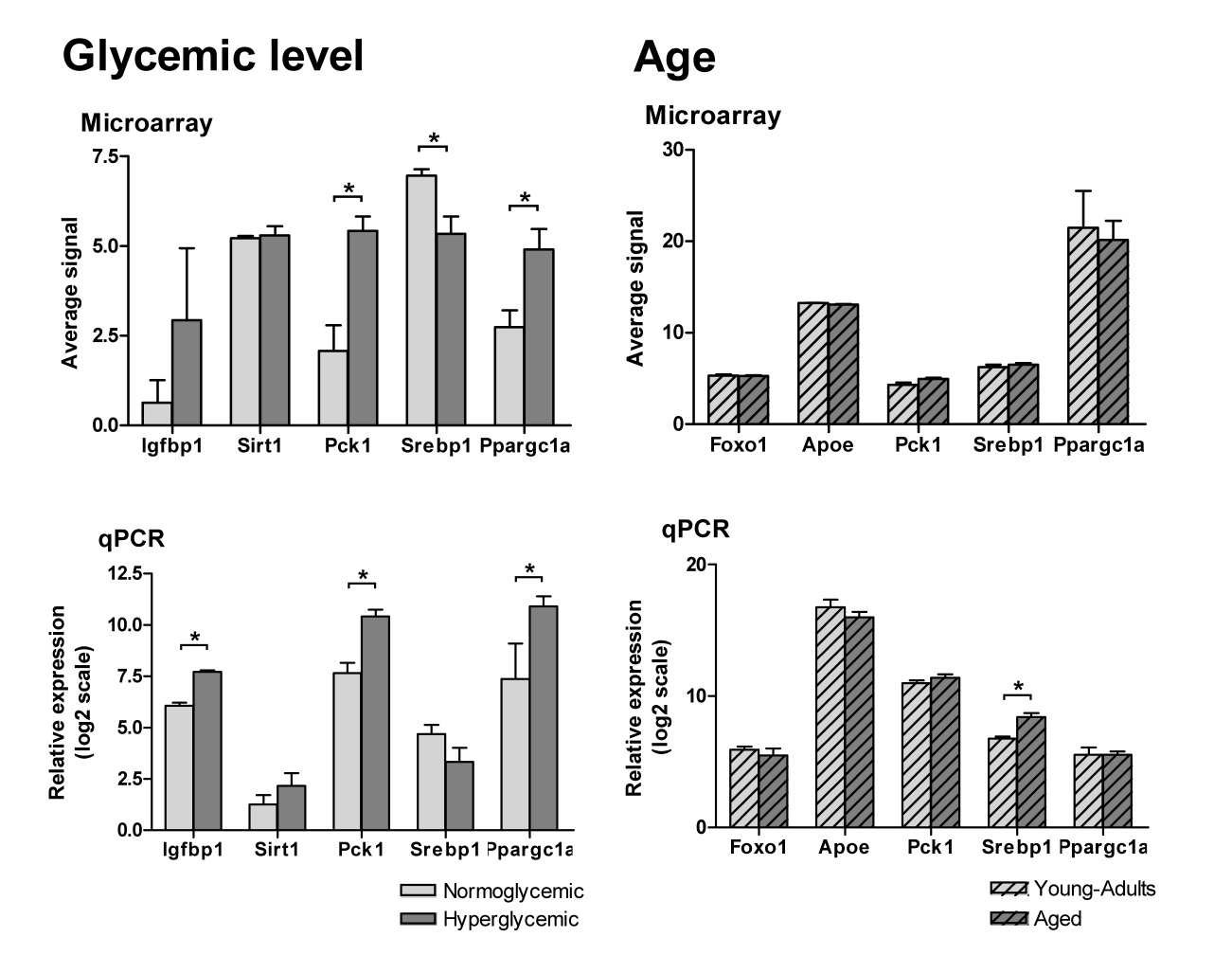
 Supplemental Table S1 – Primers used for quantitative gene expression validation.**

**Supplemental Figure S1 -** Validation of microarray results by quantitative RT-PCR. *, significative difference (P<0.05) by Student *t*-test**.**
