# Supplementary material for: Genomic profiling of type-1 adult diabetic and aged normoglycemic mouse liver
Source: BMC Endocr Disord. 2014 Mar 3;14:19. doi: 10.1186/1472-6823-14-19 (PMC4016577; doi:10.1186/1472-6823-14-19)
Supplement: Additional file 2 — List of differentially expressed genes in diabetic NOD mouse hepatocytes. [file 1472-6823-14-19-S2.docx]

**Supplemental Table S2** - Differential gene expression of diabetic mice hepatocytes.

| **Upregulated genes** | | | | | | | | | |  | | | |  |
| --- | --- | --- | --- | --- | --- | --- | --- | --- | --- | --- | --- | --- | --- | --- |
| **Gene** | **Entrez ID** | | **Fold Change** | | **p-value** | | | | |  | | | |  |
| *Transcription Regulation and Chromatin organization* | | | | | | | | | |  | | | |  |
| Bach1 | 12013 | | 5,112 | | 4,91E-03 | | | | | |  | | | |
| Ezh2 | 14056 | | 3,151 | | 1,95E-02 | | | | | |  | | | |
| Hist2h4 | 319155 | | 4,045 | | 4,20E-02 | | | | | |  | | | |
| Mbtd1 | 103537 | | 3,320 | | 3,80E-02 | | | | | |  | | | |
| Ncoa1 | 17977 | | 2,398 | | 7,29E-03 | | | | | |  | | | |
| Nr1d1 | 217166 | | 5,776 | | 3,73E-02 | | | | | |  | | | |
| Per1 | 18626 | | 3,964 | | 4,54E-02 | | | | | |  | | | |
| Ppargc1a | 19017 | | 4,487 | | 1,44E-02 | | | | | |  | | | |
| Rrp1b | 72462 | | 2,132 | | 2,41E-02 | | | | | |  | | | |
| *Metabolic process* | | | | | | | | | |  | | | |  |
| Acmsd | 266645 | | 2,626 | | 3,84E-03 | | | | | |  | | | |
| Agxt2l1 | 13178 | | 4,846 | | 9,10E-05 | | | | | |  | | | |
| Alas1 | 71760 | | 3,134 | | 2,73E-02 | | | | | |  | | | |
| Asl | 109900 | | 2,391 | | 3,21E-03 | | | | | |  | | | |
| Bhmt | 12116 | | 2,146 | | 4,38E-02 | | | | | |  | | | |
| Dusp1 | 19252 | | 2,732 | | 1,08E-02 | | | | | |  | | | |
| Entpd7 | 93685 | | 2,033 | | 3,62E-02 | | | | | |  | | | |
| Fbxo21 | 231670 | | 2,385 | | 1,19E-02 | | | | | |  | | | |
| Fem1a | 14154 | | 2,348 | | 3,60E-02 | | | | | |  | | | |
| Il12rb1 | 16161 | | 4,635 | | 3,78E-03 | | | | | |  | | | |
| Plk3 | 12795 | | 2,522 | | 1,71E-03 | | | | | |  | | | |
| *Stress Response* | | | | | | | | | | |  | | | |
| Angptl4 | 57875 | | 4,520 | | 1,43E-03 | | | | | |  | | | |
| Errfi1 | 74155 | | 2,232 | | 1,22E-02 | | | | | |  | | | |
| Fancc | 14088 | | 3,106 | | 1,25E-02 | | | | | |  | | | |
| Gadd45g | 23882 | | 2,286 | | 7,63E-03 | | | | | |  | | | |
| Lig4 | 319583 | | 2,038 | | 2,56E-02 | | | | | |  | | | |
| *Cell diferentiation* | | | | | | | | | |  | | | |  |
| Klf10 | 21847 | | 2052 | | 4,33E-02 | | | | | |  | | | |
| Klf9 | 16601 | | 2166 | | 4,78E-02 | | | | | |  | | | |
| *Lipid metabolism* | | | | | | | | | | |  | | | |
| Gde1 | 56209 | | 2,436 | | 3,62E-02 | | | | | |  | | | |
| Mogat2 | 233549 | | 3,171 | | 3,64E-03 | | | | | |  | | | |
| Npc1l1 | 237636 | | 3,057 | | 1,37E-02 | | | | | |  | | | |
| Osbpl3 | 71720 | | 2,610 | | 2,43E-02 | | | | | |  | | | |
| Scarb1 | 20778 | | 2,016 | | 2,31E-02 | | | | | | |  |  |  |
| *Transport* | | | | | | | | | | | |  |  |  |
| Rapgef4 | 56508 | | 2,580 | | 2,26E-02 | | | | | | |  |  |  |
| Scn7a | 20272 | | 2,894 | | 4,80E-02 | | | | | | |  |  |  |
| Slc17a6 | 140919 | | 5,120 | | 3,87E-04 | | | | | | |  |  |  |
| Slc28a1 | 434203 | | 3,571 | | 6,40E-03 | | | | | | |  |  |  |
| Slc37a1 | 224674 | | 3,963 | | 3,86E-03 | | | | | | |  |  |  |
| Slc37a4 | 14385 | | 2,006 | | 1,96E-02 | | | | | | |  |  |  |
| Slc7a2 | 11988 | | 4,108 | | 1,40E-02 | | | | | | |  |  |  |
| Tob1 | 22057 | | 2,253 | | 4,79E-03 | | | | | | |  |  |  |
| *Cell adhesion and motion* | | | | | | | | | | | |  |  |  |
| Cdh2 | 12558 | | 2,146 | | 1,98E-02 | | | | | | |  |  |  |
| Egfr | 13649 | | 2,220 | | 4,55E-02 | | | | | | |  |  |  |
| Epn1 | 13854 | | 2,781 | | 2,49E-02 | | | | | | |  |  |  |
| *Oxidation reduction/ respiration* | | | | | | | | | | | |  |  |  |
| Cyp17a1 | 13074 | | 3,492 | | 3,73E-03 | | | | | | |  |  |  |
| Cyp2b10 | 13088 | | 4,583 | | 3,84E-02 | | | | | | |  |  |  |
| Fh1 | 14194 | | 2,164 | | 1,38E-02 | | | | | | |  |  |  |
| *Signaling transduction* | | | | | | | | | | | |  |  |  |
| Fgf13 | 14168 | | 2,518 | | 1,02E-02 | | | | | | |  |  |  |
| Hoxd11 | 15431 | | 5,045 | | 2,28E-02 | | | | | | |  |  |  |
| Irs2 | 384783 | | 2,144 | | 4,57E-03 | | | | | | |  |  |  |
| Irs3 | 16369 | | 3,180 | | 1,69E-02 | | | | | | |  |  |  |
| Nr1d2 | 545289 | | 3,380 | | 3,73E-02 | | | | | | |  |  |  |
| Olfr411 | 258704 | | 4,012 | | 6,96E-03 | | | | | | |  |  |  |
| Olfr552 | 259106 | | 2,103 | | 4,48E-02 | | | | | | |  |  |  |
| Osmr | 18414 | | 2,187 | | 1,26E-02 | | | | | | |  |  |  |
| Rsf1 | 233532 | | 2,400 | | 4,51E-02 | | | | | | |  |  |  |
| Shpk | 74637 | | 3,112 | | 2,54E-02 | | | | | | |  |  |  |
| *Carbohydrate Metabolism* | | | | | | | | | | | |  |  |  |
| G6pc | 14377 | | 3,295 | | 6,15E-03 | | | | | | |  |  |  |
| G6pc2 | 14378 | | 3,103 | | 3,85E-02 | | | | | | |  |  |  |
| Gfpt1 | 14583 | | 2,747 | | 3,54E-02 | | | | | | |  |  |  |
| Lyg1 | 69541 | | 3,600 | | 6,16E-03 | | | | | | |  |  |  |
| Pck1 | 18534 | | 10,200 | | 3,35E-03 | | | | | | |  |  |  |
| Sds | 231691 | | 2,917 | | 1,39E-02 | | | | | | |  |  |  |
| *Immune response* | | | | | | | | | | | |  |  |  |
| Cxcl11 | 56066 | | 6,283 | | 4,56E-05 | | | | | | |  |  |  |
| Il1rl2 | 107527 | | 3,603 | | 1,61E-02 | | | | | | |  |  |  |
| Il6ra | 16194 | | 2,007 | | 4,06E-02 | | | | | | |  |  |  |
| Saa3 | 20210 | | 2,215 | | 3,22E-02 | | | |  | | | |  |  |
| Tlr11 | 239081 | | 3,370 | | 1,95E-03 | | | |  | | | |  |  |
| Ung | 22256 | | 3,483 | | 2,80E-03 | | | |  | | | |  |  |
| *Miscelaneous* | | | | | | | | |  | | | |  |  |
| Ccrn4l | 12457 | | 3,121 | | 1,71E-02 | | | |  | | | |  |  |
| Cep110 | 26920 | | 2,346 | | 2,25E-02 | | | |  | | | |  |  |
| Coq10b | 67876 | | 2,504 | | 4,28E-03 | | | |  | | | |  |  |
| Dck | 13178 | | 3,531 | | 3,06E-02 | | | |  | | | |  |  |
| Eif4ebp3 | 100128771 | | 9,316 | | 3,58E-02 | | | |  | | | |  |  |
| Gas5 | 14455 | | 2,389 | | 9,50E-03 | | | |  | | | |  |  |
| Gzmk | 14945 | | 3,905 | | 1,31E-02 | | | |  | | | |  |  |
| Igsf5 | 72058 | | 2,363 | | 1,25E-02 | | | |  | | | |  |  |
| Luzp2 | 233271 | | 2,393 | | 1,57E-02 | | | | | | |  |  |  |
| Mex3c | 240396 | | 2,504 | | 7,80E-03 | | | | | | |  |  |  |
| Nnmt | 18113 | | 9,727 | | 3,52E-03 | | | | | | |  |  |  |
| Pim3 | 223775 | | 3,118 | | 3,37E-02 | | | | | | |  |  |  |
| Pptc7 | 320717 | | 2,336 | | 3,83E-02 | | | | | | |  |  |  |
| Ren1 | 100044656 | | 3,623 | | 1,09E-02 | | | | | | |  |  |  |
| Rnf149 | 67702 | | 3,343 | | 1,23E-03 | | | | | | |  |  |  |
| Rpusd1 | 106707 | | 2,905 | | 2,10E-02 | | | | | | |  |  |  |
| Sh3d19 | 27059 | | 2,612 | | 1,91E-02 | | | | | | |  |  |  |
| Susd4 | 96935 | | 3,659 | | 4,45E-03 | | | | | | |  |  |  |
| **Downregulated genes** | | | | | | | |  | | | | | | |
| **Gene** | | **Entrez ID** | | **Fold Change** | **p-value** | | |  | | | | | | |
| *Integral to membrane* | | | | | | | |  | | | | | | |
| Adam28 | | 13522 | | -2,490 | 2,33E-02 | | |  | | | | | | |
| Armcx3 | | 71703 | | -2,044 | 4,56E-02 | | |  | | | | | | |
| B3galt5 | | 93961 | | -3,039 | 2,44E-02 | | |  | | | | | | |
| Bst2 | | 69550 | | -2,942 | 1,01E-02 | | |  | | | | | | |
| Cd2 | | 12481 | | -2,196 | 2,49E-02 | | |  | | | | | | |
| Cldn1 | | 12737 | | -2,298 | 4,05E-02 | | |  | | | | | | |
| Gja5 | | 14613 | | -2,474 | 3,21E-02 | | |  | | | | | | |
| Klrd1 | | 16643 | | -4,902 | 2,19E-03 | | |  | | | | | | |
| Mme | | 17380 | | -2,815 | 4,60E-02 | | |  | | | | | | |
| Mrgpre | | 244238 | | -5,023 | 4,51E-03 | | |  | | | | | | |
| Pdzk1ip1 | | 67182 | | -6,599 | 2,16E-02 | | | |  | | | | | |
| Pvrl1 | | 58235 | | -3,391 | 7,25E-03 | | | |  | | | | | |
| Reep1 | | 52250 | | -5,608 | 2,15E-02 | | | |  | | | | | |
| Scfd1 | | 76983 | | -2,031 | 1,53E-02 | | | |  | | | | | |
| Siglec1 | | 20612 | | -4,481 | 4,34E-02 | | | |  | | | | | |
| Slc41a1 | | 98396 | | -6,154 | 2,24E-03 | | | |  | | | | | |
| Slc44a1 | | 100434 | | -2,287 | 1,99E-02 | | | |  | | | | | |
| Slc7a8 | | 50934 | | -3,116 | 3,93E-02 | | | |  | | | | | |
| Sucnr1 | | 84112 | | -5,961 | 2,57E-02 | | | |  | | | | | |
| Tmem144 | | 70652 | | -4,573 | 1,63E-04 | | | |  | | | | | |
| *Phosphate metabolic process* | | | | | | | |  | | | | | | |
| Eef2k | | 13631 | | -9,352 | 1,23E-03 | | | |  | | | | | |
| Lrrk2 | | 66725 | | -2,511 | 2,12E-02 | | | |  | | | | | |
| Nek6 | | 59126 | | -3,117 | 1,69E-02 | | | |  | | | | | |
| Pbk | | 52033 | | -3,850 | 3,54E-03 | | | | | | | | | |
| Rapgef3 | | 2223864 | | -2,343 | 6,25E-03 | | | | | | | | | |
| *Regulation of transcription* | | | | | | | | | | | | | | |
| Atf6 | | 226641 | | -2,331 | 8,60E-03 | | | | | | | | | |
| Csrp2bp | | 228714 | | -2,015 | 4,36E-02 | | | | | | | | | |
| Dlgap1 | | 224997 | | -2,044 | 4,88E-02 | | | | | | | | | |
| Ebf1 | | 13591 | | -3,901 | 2,23E-02 | | | | | | | | | |
| Eid1 | | 58521 | | -2,693 | 2,13E-03 | | | | | | | | | |
| Eif2c4 | | 76850 | | -2,423 | 3,03E-02 | | | | | | | | | |
| Hltf | | 20585 | | -2,300 | 1,15E-02 | | | | | | | | | |
| Maml1 | | 103806 | | -5,163 | 2,45E-02 | | | | | | | | | |
| Mbd6 | | 110962 | | -2,433 | 2,41E-02 | | | | | | | | | |
| Npas2 | | 18143 | | -2,005 | 3,93E-02 | | | | | | | | | |
| Pdgfc | | 54635 | | -2,201 | 1,44E-02 | | | | | | | | | |
| Tcf12 | | 21406 | | -2,414 | 4,66E-02 | | | | | | | | | |
| Zbtb46 | | 72147 | | -3,788 | 3,26E-02 | | | | | | | | | |
| *Response to stress* | | | | | | | | | | | | | | |
| Abp1 | | 76507 | | -4,383 | 3,64E-02 | | | | | | | | | |
| Apex1 | | 11792 | | -2,082 | 3,29E-02 | | | | | | | | | |
| Bbc3 | | 170770 | | -2,088 | 2,25E-02 | | | | | | | | | |
| Btc | | 12223 | | -6,343 | 1,72E-04 | | | | | | | | | |
| Casp3 | | 12367 | | -2,979 | 8,26E-03 | | | | | | | | | |
| Cav1 | | 12389 | | -3,711 | 4,99E-02 | | | | | | | | | |
| Ccnf | | 12449 | | -2,587 | 2,16E-02 | | | | | | | | | |
| Cd44 | | 12505 | | -2,416 | 3,68E-03 | | | | | | | | | |
| Cdc123 | | 98828 | | -3,926 | 1,38E-02 | | | | | | | | | |
| Colec10 | | 239447 | | -3,859 | 1,34E-02 | | | | | | | | | |
| Cyr61 | | 16007 | | -2,836 | 1,90E-02 | | | | | | | | | |
| Elmod2 | | 244548 | | -2,984 | 1,98E-02 | |  | | | | | | | |
| Gas6 | | 14456 | | -3,481 | 7,27E-03 | |  | | | | | | | |
| Huwe1 | | 59026 | | -2,028 | 4,84E-02 | |  | | | | | | | |
| Itgb3 | | 16416 | | -3,368 | 2,24E-02 | |  | | | | | | | |
| Klk1b4 | | 18048 | | -7,994 | 2,76E-03 | |  | | | | | | | |
| Lck | | 16818 | | -2,274 | 1,23E-02 | |  | | | | | | | |
| Met | | 17295 | | -4,508 | 3,37E-02 | |  | | | | | | | |
| Pdia2 | | 69191 | | -4,001 | 2,44E-03 | |  | | | | | | | |
| Sfrp5 | | 54612 | | -2,044 | 3,02E-02 | |  | | | | | | | |
| Tnfrsf14 | | 230979 | | -6,136 | 2,09E-04 | |  | | | | | | | |
| *Immune response* | | | | | | |  | | | | | | | |
| C8a | | 230558 | | -2,411 | 7,17E-03 | |  | | | | | | | |
| ccl21 | | 18829 | | -4,124 | 5,54E-03 | |  | | | | | | | |
| Ccl24 | | 56221 | | -2,220 | 6,02E-03 | |  | | | | | | | |
| Cd276 | | 102657 | | -5,451 | 1,17E-02 | |  | | | | | | | |
| Cd44 | | 12505 | | -2,416 | 3,68E-03 | |  | | | | | | | |
| Fasl | | 14103 | | -4,200 | 4,71E-03 | |  | | | | | | | |
| H2-Q10 | | 15007 | | -5,439 | 3,08E-04 | |  | | | | | | | |
| Il2rg | | 16186 | | -3,831 | 6,27E-03 | |  | | | | | | | |
| Irf7 | | 54123 | | -5,610 | 7,73E-03 | |  | | | | | | | |
| Ndrg1 | | 17988 | | -2,271 | 3,14E-02 | |  | | | | | | | |
| Nkx2-3 | | 18089 | | -3,150 | 1,64E-02 | |  | | | | | | | |
| Oas2 | | 246728 | | -9,183 | 1,78E-04 | |  | | | | | | | |
| *Carbohydrate Metabolism* | | | | | |  | | | | | | | | |
| G6pdx | | 14381 | | -2,002 | 3,10E-02 | |  | | | | | | | |
| Gck | | 103988 | | -3,535 | 9,88E-03 | |  | | | | | | | |
| Hsd3b5 | | 15496 | | -21,604 | 4,07E-04 | |  | | | | | | | |
| *Lipid Metabolism* | | | | | |  | | | | | | | | |
| Aacs | | 78894 | | -2,616 | 2,87E-02 | |  | | | | | | | |
| Aadac | | 67758 | | -6,324 | 2,96E-02 | |  | | | | | | | |
| Acacb | | 100705 | | -2,598 | 2,65E-02 | |  | | | | | | | |
| Fabp5 | | 16592 | | -12,945 | 5,80E-05 | |  | | | | | | | |
| Gpam | | 14732 | | -3,964 | 3,44E-02 | |  | | | | | | | |
| Hsd17b2 | | 15486 | | -2,444 | 3,42E-02 | |  | | | | | | | |
| Oxct1 | | 67041 | | -2,336 | 3,33E-02 | |  | | | | | | | |
| Plp1 | | 18823 | | -3,814 | 5,86E-03 | |  | | | | | | | |
| Pnpla3 | | 116939 | | -27,097 | 1,78E-06 | |  | | | | | | | |
| Pnpla5 | | 75772 | | -34,746 | 1,85E-05 | |  | | | | | | | |
| Ppard | | 19015 | | -3,300 | 6,95E-03 | |  | | | | | | | |
| Soat2 | | 223920 | | -2,332 | 4,24E-02 | |  | | | | | | | |
| Srebf1 | | 20787 | | -3,081 | 1,38E-02 | |  | | | | | | | |
| *Extracellular Space* | | | | | | | | | | | | | | |
| Col6a3 | | 12835 | | -2,081 | 4,08E-02 | | | | | | | | | |
| Fbln1 | | 14114 | | -2,717 | 1,45E-02 | | | | | | | | | |
| Ifna13 | | 230396 | | -2,222 | 4,00E-02 | | | | | | | | | |
| Mup3 | | 17842 | | -2,209 | 2,06E-02 | | | | | | | | | |
| Spt1 | | 20770 | | -2,082 | 4,87E-02 | | | | | | | | | |
| *Cytoskeleton organization* | | | | | | | | | | | | | | |
| Acta2 | | 11475 | | -2027 | 1,92E-02 | | | | | | | | | |
| Bmp5 | | 12160 | | -3,348 | 9,17E-03 | | | | | | | | | |
| Cckar | | 12425 | | -2,979 | 4,97E-02 | | | | | | | | | |
| Dctn1 | | 13191 | | -2,510 | 6,39E-03 | | | | | | | | | |
| Dynll2 | | 68097 | | -2,042 | 3,01E-02 | | | | | | | | | |
| Rassf3 | | 192678 | | -2,260 | 4,41E-02 | | | | | | | | | |
| Svil | | 225115 | | -2,153 | 3,71E-02 | | | | | | | | | |
| *Metabolic processes* | | | | | | | | | | | | | | |
| Amd2 | | 100041585 | | -4,177 | 3,80E-02 | | | | | | | | | |
| Cacybp | | 12301 | | -5,510 | 6,55E-04 | | | | | | | | | |
| Itln1 | | 16429 | | -3,337 | 8,20E-03 | | | | | | | | | |
| Moxd1 | | 59012 | | -2,750 | 1,77E-03 | | | | | | | | | |
| Npr3 | | 18162 | | -2,189 | 4,19E-02 | | | | | | | | | |
| *Signal transduction* | | | | | | | | | | | | | | |
| Arrdc3 | | 105171 | | -2,428 | 3,54E-02 | | | | | | | | | |
| Epgn | | 71920 | | -2,698 | 4,31E-02 | | | | | | | | | |
| Esr1 | | 13982 | | -2,011 | 8,69E-03 | | | | | | | | | |
| Git1 | | 216963 | | -2,475 | 4,47E-02 | | | | | | | | | |
| Ltbp4 | | 108075 | | -2,015 | 5,20E-03 | | | | | | | | | |
| Plxna2 | | 18845 | | -2,553 | 4,89E-02 | | | | | | | | | |
| Rab38 | | 72433 | | -4,157 | 9,77E-03 | | | | | | | | | |
| Wfikkn2 | | 278507 | | -2,477 | 1,04E-02 | | | | | | | | | |
| *Miscelaneous* | | | | | | | | | | | | | | |
| Apcs | | 20219 | | -2,284 | 1,72E-02 | | | | | | | | | |
| Car2 | | 12349 | | -4,060 | 1,39E-03 | | | | | | | | | |
| Cox6b2 | | 333182 | | -2,205 | 4,61E-02 | | | | | | | | | |
| Dut | | 110074 | | -2,932 | 8,40E-03 | | | | | | | | | |
| Entpd6 | | 12497 | | -3,242 | 4,08E-02 | | | | | | | | | |
| Gm97 | | 225923 | | -2,240 | 4,92E-02 | | | | | | | | | |
| Klhdc9 | | 68874 | | -2,735 | 4,44E-02 | | | | | | | | | |
| Klhl26 | | 234378 | | -2,031 | 4,18E-03 | | | | | | | | | |
| Lin37 | | 75660 | | -3,856 | 2,01E-02 | | | | | | | | | |
| Lrriq3 | | 74435 | | -5,544 | 1,19E-02 | | | | | | | | | |
| Mst1 | | 15235 | | -2,556 | 2,65E-02 | | | | | | | | | |
| Mug2 | | 17837 | | -3,781 | 1,41E-02 | | | | | | | | | |
| Olfr110 | | 258325 | | -2,923 | 2,12E-02 | | | |  | | | | | |
| Sema6a | | 20358 | | -2,164 | 8,43E-03 | | | |  | | | | | |
| Strn | | 268980 | | -2,829 | 1,88E-02 | | | |  | | | | | |
| Trip13 | | 69716 | | -2,678 | 4,71E-02 | | | | | | | | | |
| Ugt1a2 | | 22236 | | -2,018 | 4,77E-02 | | | | | | | | | |
