# Supplementary material for: Genomic profiling of type-1 adult diabetic and aged normoglycemic mouse liver
Source: BMC Endocr Disord. 2014 Mar 3;14:19. doi: 10.1186/1472-6823-14-19 (PMC4016577; doi:10.1186/1472-6823-14-19)
Supplement: Additional file 3 — List of differentially expressed genes in middle-aged Balb/c mouse hepatocytes. [file 1472-6823-14-19-S3.docx]

Supplemental Table S3 - Differential gene expression of middle-aged mice hepatocyte.

| **Upregulated genes** | | | | |  | | |
| --- | --- | --- | --- | --- | --- | --- | --- |
| **Gene** | **Entrez ID** | **Fold Change** | **p-value** | |  | | |
| *Lipid Metabolism* | | | | |  | | |
| Acot4 | 171282 | 2,022 | 3,85E-03 | |  | | |
| Agpat1 | 55979 | 2,166 | 1,61E-02 | |  | | |
| B4galt6 | 56386 | 3,091 | 2,02E-02 | |  | | |
| Cel | 12613 | 320,576 | 1,11E-03 | |  | | |
| Fabp3 | 14077 | 10,132 | 1,89E-02 | |  | | |
| Nampt | 59027 | 2,414 | 4,39E-02 | |  | | |
| Pla2g1b | 18778 | 116,197 | 2,29E-03 | |  | | |
| Pnlip | 69060 | 1428,803 | 1,09E-04 | |  | | |
| Slc27a4 | 26569 | 2,219 | 6,52E-03 | |  | | |
| Sult2a1 | 20859 | 3,404 | 1,45E-02 | |  | | |
| Vldlr | 22359 | 2,875 | 9,07E-03 | |  | | |
| *Transport* | | | | |  | | |
| Abcc4 | 239273 | 4,930 | 2,41E-04 | |  | | |
| Atp2a3 | 53313 | 2,635 | 9,91E-03 | |  | | |
| Kcnk13 | 217826 | 3,144 | 3,02E-02 | |  | | |
| Ltf | 17002 | 2,371 | 2,54E-02 | |  | | |
| Scnn1g | 20278 | 3,206 | 5,09E-03 | |  | | |
| Slc13a3 | 114644 | 2,205 | 1,69E-02 | |  | | |
| Slc22a17 | 59049 | 2,741 | 3,86E-02 | |  | | |
| Slc8a1 | 20541 | 2,419 | 3,64E-02 | |  | | |
| Snx3 | 54198 | 2,639 | 1,91E-02 | |  | | |
| Trpm7 | 58800 | 2,622 | 4,91E-03 | |  | | |
| *Imune response* | | | | |  | | |
| Ccl5 | 20304 | 3,529 | 2,60E-02 | |  | | |
| Cd83 | 12522 | 2,649 | 2,86E-02 | |  | | |
| Cfd | 11537 | 6,323 | 1,32E-03 | |  | | |
| H2-Bl | 14963 | 2,264 | 3,94E-02 | |  | | |
| H2-DMb2 | 15000 | 3,149 | 2,78E-02 | |  | | |
| Ifna1 | 15962 | 2,378 | 1,53E-03 | |  | | |
| Reg3a | 19694 | 3,574 | 3,50E-03 | |  | | |
| Tlr1 | 21897 | 2,042 | 1,03E-02 | |  | | |
| *Regulation of transcription* | | | | |  | | |
| Ar | 18835 | 4,369 | 9,72E-04 | |  | | |
| Cux2 | 13048 | 2,945 | 1,08E-02 | |  | | |
| Dbp | 13170 | 63,161 | 6,79E-06 | |  | | |
| Egr1 | 13653 | 3,023 | 2,43E-02 | |  | | |
| Esrrg | 26381 | 3,374 | 1,55E-03 | | |  |  |
| Mcm10 | 70024 | 2,044 | 4,19E-03 | | |  |  |
| Nr1d1 | 217166 | 20,445 | 5,15E-06 | | |  |  |
| Nr1d2 | 353187 | 11,381 | 4,52E-03 | | |  |  |
| Per2 | 18627 | 3,079 | 5,86E-03 | | |  |  |
| Per3 | 18628 | 21,345 | 3,59E-06 | | |  |  |
| Pou2af1 | 18985 | 5,827 | 1,14E-03 | | |  |  |
| Sp2 | 78912 | 3,257 | 1,39E-03 | | |  |  |
| Tnni2 | 21953 | 26,947 | 4,12E-02 | | |  |  |
| Zfp608 | 269023 | 3,102 | 3,45E-03 | | |  |  |
| *Signal Transduction* | | | | | |  |  |
| Arrdc3 | 105171 | 2,856 | 3,26E-03 | | |  |  |
| Ccrn4l | 12457 | 3,084 | 1,45E-03 | | |  |  |
| Dlgap1 | 224997 | 2,220 | 1,39E-02 | | |  |  |
| Egf | 13645 | 2,947 | 2,47E-02 | | |  |  |
| Fgf21 | 56636 | 2,215 | 3,38E-02 | | |  |  |
| Kiss1 | 280287 | 2,121 | 1,37E-02 | | |  |  |
| Lime1 | 72699 | 5,676 | 1,76E-02 | | |  |  |
| Ly6c1 | 17067 | 2,465 | 1,04E-02 | | |  |  |
| Olfr148 | 258498 | 2,894 | 1,85E-03 | | |  |  |
| Pdlim4 | 30794 | 2,330 | 4,20E-03 | | |  |  |
| Rab30 | 75985 | 2,345 | 4,09E-02 | | |  |  |
| Rgs16 | 19734 | 13,488 | 5,77E-04 | | |  |  |
| Stmn2 | 20257 | 3,176 | 2,78E-02 | | |  |  |
| Sucnr1 | 84112 | 4,945 | 3,81E-04 | | |  |  |
| Tbc1d10a | 103724 | 2,498 | 4,97E-02 | | |  |  |
| *Response to stress/ cell division* | | | | | |  |  |
| Bcl2a1d | 12047 | 2,578 | 2,61E-03 | | |  |  |
| Ccnk | 12454 | 2,914 | 1,22E-02 | | |  |  |
| Cd3g | 12502 | 2,052 | 2,74E-02 | | |  |  |
| Chac1 | 69065 | 9,966 | 1,77E-02 | | |  |  |
| Elmo2 | 140579 | 2,213 | 1,68E-03 | | |  |  |
| Gadd45a | 13197 | 5,510 | 1,48E-03 | | |  |  |
| Gas2l3 | 237436 | 3,601 | 2,44E-02 | | |  |  |
| Hspb1 | 15507 | 3,648 | 3,62E-03 | | |  |  |
| Mcm6 | 17219 | 2,075 | 4,58E-02 | | |  |  |
| Nek6 | 59126 | 2,297 | 3,43E-02 | | |  |  |
| Nqo1 | 18104 | 2,029 | 3,68E-03 | | |  |  |
| Nupr1 | 56312 | 5,180 | 2,68E-02 | | |  |  |
| Ogg1 | 18294 | 2,096 | 1,70E-02 | | |  |  |
| Pdia2 | 69191 | 24,671 | 3,00E-02 |  | | |  |
| Pim3 | 22375 | 2,003 | 4,25E-03 |  | | |  |
| Prkce | 18754 | 2,713 | 2,15E-03 |  | | |  |
| *Oxidation Reduction* | | | |  | | |  |
| Cox6a2 | 12862 | 23,487 | 1,08E-02 |  | | |  |
| Cyp2a4 | 13086 | 2,527 | 1,20E-03 |  | | |  |
| Cyp2c40 | 545288 | 3,489 | 3,16E-03 |  | | |  |
| Cyp46a1 | 13116 | 3,900 | 2,73E-02 |  | | |  |
| Cyp4a14 | 13119 | 3,052 | 6,59E-03 |  | | |  |
| Por | 18984 | 2,158 | 4,94E-03 |  | | |  |
| *Development* | | | |  | | |  |
| Acrbp | 54137 | 2,764 | 4,99E-03 |  | | |  |
| Amigo1 | 229715 | 3,878 | 4,54E-02 |  | | |  |
| Ank3 | 11735 | 2,022 | 1,81E-02 |  | | |  |
| Aoc3 | 11754 | 3,814 | 4,76E-04 |  | | |  |
| Arvcf | 11877 | 2,333 | 2,55E-02 |  | | |  |
| Col12a1 | 12816 | 2,082 | 1,99E-02 |  | | |  |
| Cytip | 227929 | 2,334 | 5,81E-04 |  | | |  |
| Trip13 | 69716 | 3,039 | 2,07E-03 |  | | |  |
| *Cell Adhesion* | | | |  | | |  |
| Hexb | 15212 | 2,060 | 1,37E-02 |  | | |  |
| Ntrk2 | 18212 | 3,301 | 4,20E-02 |  | | |  |
| Tef | 21685 | 3,943 | 2,20E-04 |  | | |  |
| Thbs2 | 21826 | 2,283 | 1,33E-02 |  | | |  |
| Usp2 | 53376 | 2,951 | 3,06E-02 |  | | |  |
| *Proteins Integral of Membrane* | | | |  | | |  |
| Coq10b | 67876 | 2,191 | 5,48E-03 |  | | |  |
| Fam18b | 67510 | 4,220 | 2,34E-04 |  | | |  |
| Gnptab | 432486 | 2,072 | 4,10E-02 | | |  |  |
| Il17rd | 171463 | 2,494 | 4,35E-02 | | |  |  |
| Plscr2 | 18828 | 2,074 | 4,06E-02 | | |  |  |
| Xlr5c | 27084 | 2,541 | 4,83E-02 | | |  |  |
| *Miscelaneous* | | | | | |  |  |
| Adamdec1 | 58860 | 3,332 | 3,50E-02 | | |  |  |
| Angel1 | 68737 | 5,710 | 5,94E-04 | | |  |  |
| Arhgap26 | 71302 | 2,565 | 3,64E-02 | | |  |  |
| Cnpy1 | 269637 | 2,719 | 2,68E-02 | | |  |  |
| Ctrl | 109660 | 164,780 | 1,78E-03 | | |  |  |
| Eif5b | 226982 | 3,447 | 1,29E-04 | | |  |  |
| Endod1 | 71946 | 2,290 | 8,03E-04 | | |  |  |
| Fmn2 | 54418 | 2,283 | 3,08E-03 | | |  |  |
| Gpc1 | 14733 | 2,831 | 1,51E-03 | | |  |  |
| Kif13a | 16553 | 2,044 | 4,08E-02 | | |  |  |
| Klk1 | 16612 | 41,587 | 8,37E-03 | | |  |  |
| Lalba | 16770 | 2,564 | 3,75E-02 | | |  |  |
| Mid1 | 17318 | 3,103 | 9,48E-03 | | |  |  |
| Mylk2 | 228785 | 2,363 | 4,05E-03 | | |  |  |
| Pcsk4 | 18551 | 3,099 | 1,23E-03 | | |  |  |
| Reg2 | 19693 | 11,212 | 7,12E-03 | | |  |  |
| Rftn1 | 76438 | 5,129 | 4,06E-04 | | |  |  |
| Scpep1 | 74617 | 2,841 | 5,10E-04 | | |  |  |
| Serpinb1a | 66222 | 5,626 | 2,42E-03 | | |  |  |
| Smurf1 | 75788 | 3,068 | 1,15E-02 | | |  |  |
| Trem1 | 58217 | 3,546 | 2,67E-02 | | |  |  |
| Try4 | 22074 | 565,093 | 3,55E-04 | | |  |  |
| Zfp217 | 228913 | 4,973 | 1,58E-04 | | |  |  |

| **Downregulated genes** | | | |  | | |  |
| --- | --- | --- | --- | --- | --- | --- | --- |
| **Gene** | **Entrez ID** | **Fold Change** | **p-value** |  | | |  |
| *Post-translational modification* | | | |  | | |  |
| Eif4ebp3 | 1088112 | -5,532 | 6,42E-04 |  | | |  |
| Lonrf3 | 74365 | -2,425 | 2,29E-03 |  | | |  |
| Rpl22l1 | 68028 | -5,972 | 1,89E-04 |  | | |  |
| *Chromatin structure and dynamic* | | | |  | | |  |
| Dnmt3b | 13436 | -2,003 | 2,39E-02 |  | | |  |
| Hist1h4c | 319155 | -3,016 | 5,78E-03 |  | | |  |
| Smarcd1 | 83797 | -4,257 | 2,98E-04 | | |  |  |
| Ubn1 | 170644 | -2,461 | 6,63E-03 | | |  |  |
| *Lipid metabolism* | | | | | |  |  |
| Acacb | 100705 | -2,210 | 2,84E-02 | | |  |  |
| Acot6 | 217700 | -4,211 | 5,72E-04 | | |  |  |
| Akr1c21 | 77337 | -2,741 | 3,57E-02 | | |  |  |
| Akr1d1 | 208665 | -4,158 | 6,80E-03 | | |  |  |
| Hsd3b5 | 15496 | -12,560 | 1,06E-02 | | |  |  |
| Ptgds | 19215 | -4,186 | 7,70E-03 | | |  |  |
| *Oxidation Reduction* | | | | | |  |  |
| Cyp2b10 | 13088 | -4,178 | 7,27E-04 | |  | | |
| Cyp2c54 | 404195 | -2,068 | 4,56E-02 | |  | | |
| Cyp2d9 | 13105 | -3,664 | 2,95E-03 | |  | | |
| Cyp3a11 | 13112 | -2,291 | 1,37E-03 | |  | | |
| Mical2 | 320878 | -3,116 | 3,09E-02 | |  | | |
| *Carbohydrate metabolism* | | | | |  | | |
| B3gnt8 | 232984 | -2,547 | 4,16E-02 | |  | | |
| G6pdx | 14381 | -2,378 | 5,20E-03 | |  | | |
| Ppp1r3c | 53412 | -2,759 | 1,47E-03 | |  | | |
| Serpina4-ps1 | 321018 | -9,642 | 7,35E-03 | |  | | |
| *Signal transduction* | | | | |  | | |
| Adam24 | 13526 | -2,697 | 1,97E-02 | |  | | |
| Adam28 | 13522 | -2,091 | 1,69E-02 | |  | | |
| Akt1 | 11651 | -6,912 | 1,20E-04 | |  | | |
| Gpr65 | 14744 | -2,049 | 3,92E-02 | |  | | |
| Ighmbp2 | 20589 | -2,041 | 4,77E-02 | |  | | |
| Mt2 | 117750 | -4,463 | 3,13E-02 | |  | | |
| Olfr273 | 258821 | -2,381 | 3,83E-02 | |  | | |
| Olfr341 | 258952 | -2,335 | 3,12E-02 | |  | | |
| Olfr914 | 258782 | -2,028 | 3,39E-02 | |  | | |
| Plekhg6 | 213522 | -2,265 | 2,41E-02 | |  | | |
| Rgs13 | 246709 | -3,949 | 2,63E-03 | |  | | |
| Trh | 22044 | -3,457 | 4,00E-03 | |  | | |
| Trp53rk | 76367 | -3,411 | 1,55E-02 | |  | | |
| Wnt3a | 22416 | -4,080 | 4,70E-03 | |  | | |
| *Miscelaneous* | | | | |  | | |
| Abca12 | 74591 | -2,307 | 4,90E-02 | |  | | |
| Cd209f | 69142 | -2,499 | 1,00E-03 | |  | | |
| Fez1 | 235180 | -2,970 | 7,10E-03 | |  | | |
| Gan | 209239 | -2,375 | 1,35E-02 | |  | | |
| Lama5 | 16776 | -2,369 | 3,31E-02 | |  | | |
| Plxdc2 | 67448 | -2,720 | 6,48E-03 | |  | | |
| Serpina9 | 71907 | -5,944 | 1,84E-04 | |  | | |
| Susd4 | 96935 | -2,787 | 1,94E-02 | |  | | |
| Tmem35 | 67564 | -2,269 | 2,64E-02 | |  | | |
| Tubb2b | 73710 | -2,307 | 1,00E-03 | |  | | |
| Zmynd10 | 114602 | -2,661 | 1,13E-02 | |  | | |
| *Transcription* | | | | | |  |  |
| Irak1bp1 | 65099 | -3,445 | 4,64E-02 | | |  |  |
| Nat14 | 269854 | -4,437 | 4,83E-03 | | |  |  |
| Neurog3 | 11925 | -2,353 | 4,28E-02 | | |  |  |
| Npas2 | 18143 | -8,986 | 6,12E-03 | | |  |  |
| Ppard | 19015 | -6,500 | 3,42E-04 | | |  |  |
| Ppp1r10 | 52040 | -2,046 | 2,32E-02 | | |  |  |
| Zbtb7a | 16969 | -2,471 | 1,37E-02 | | |  |  |
| *Response to stress* | | | | | |  |  |
| Rif1 | 51869 | -2,721 | 7,09E-03 | | |  |  |
| Sncb | 104069 | -2,466 | 1,51E-02 | | |  |  |
| *Transport* | | | | | |  |  |
| Aqp8 | 11833 | -2,155 | 1,95E-03 | | |  |  |
| Slc25a18 | 71803 | -2,703 | 3,11E-03 | | |  |  |
| Slc25a22 | 68267 | -3,049 | 4,79E-02 | | |  |  |
| Slc34a2 | 20531 | -4,195 | 3,70E-02 | | |  |  |
| Slc39a5 | 72002 | -2,149 | 1,75E-02 | | |  |  |
| Trpv5 | 194352 | -2,589 | 3,40E-02 | | |  |  |
| *Inflammatory response* | | | | | |  |  |
| C8b | 110382 | -3,257 | 1,75E-03 | | |  |  |
| Ccl20 | 20297 | -2,855 | 1,29E-03 | | |  |  |
| Cdkn1a | 12575 | -4,019 | 2,24E-02 | | |  |  |
| Cx3cr1 | 13051 | -2,039 | 3,80E-02 | | |  |  |
| Il1r1 | 16177 | -3,359 | 2,27E-02 | | |  |  |
| Shpk | 74637 | -2,779 | 4,86E-02 | | |  |  |
| *Cell Adhesion* | | | | | |  |  |
| Cdh1 | 12550 | -2,125 | 2,02E-02 | | |  |  |
| Igsf5 | 72058 | -2,857 | 3,78E-02 | | |  |  |
| Jam2 | 67374 | -2,037 | 1,39E-02 | | |  |  |
| Lrfn3 | 233067 | -2,398 | 9,01E-03 | | |  |  |
| *Development* | | | | | |  |  |
| Egfr | 13649 | -3,050 | 2,28E-03 | | |  |  |
| Gdf11 | 14561 | -2,205 | 1,69E-02 | | |  |  |
| Mmp19 | 58223 | -2,681 | 1,84E-02 | | |  |  |
| Tnfrsf11a | 21934 | -2,282 | 3,14E-02 | | |  |  |
| Zbtb16 | 235320 | -4,818 | 5,98E-03 | | |  |  |
